# Hospital Pharmacists’ Viewpoint on Quality Use of Antibiotics and Resistance: A Qualitative Exploration from a Tertiary Care Hospital of Quetta City, Pakistan

**DOI:** 10.3390/antibiotics12081343

**Published:** 2023-08-21

**Authors:** Maryam Farooqui, Zaffar Iqbal, Abdul Sadiq, Abdul Raziq, Mohammed Salem Alshammari, Qaiser Iqbal, Sajjad Haider, Fahad Saleem

**Affiliations:** 1 Department of Pharmacy Practice, Unaizah College of Pharmacy, Qassim University, Buraydah 52571, Saudi Arabia; m.farooqui@qu.edu.sa (M.F.); m.alshammari@qu.edu.sa (M.S.A.); 2 Health Department, Government of Balochistan, Quetta 87100, Pakistan; zaffar_khosti@ymail.com; 3 Jhalawan Medical College Khuzdar, Khuzdar 89100, Pakistan; drabdulsadiq@gmail.com; 4 Department of Statistics, University of Balochistan, Quetta 87300, Pakistan; raziq@um.uob.edu.pk; 5 Faculty of Pharmacy & Health Sciences, University of Balochistan, Quetta 87300, Pakistan; qaiser.pharm@um.uob.edu.pk (Q.I.); sajjad.pharm@um.uob.edu.pk (S.H.)

**Keywords:** antibiotic use, antibiotics resistance, hospital pharmacists, qualitative, Quetta city, Pakistan

## Abstract

Suboptimal antibiotics use and the development of antibiotic resistance is a universal calamity. The theoretical model of therapeutic efficacy correlates quality use of antibiotics with healthcare practitioners’ understanding of antibiotic use and resistance. Keeping this phenomenon in mind, we aimed to evaluate hospital pharmacists’ understanding of antibiotic use and resistance at a public healthcare institute in Quetta city, Pakistan. This was a qualitative study that employed a semi-structured interview guide for data extraction. The phenomenology-based approach commissioned in-depth, face-to-face interviews with hospital pharmacists stationed at the surgical unit of Sandeman Provincial Hospital, Quetta. The interviews were audio taped followed by transcribed verbatim and were then analyzed for thematic contents by the standard content analysis framework. Although the saturation was reached after the 10th interview, we conducted two additional interviews for definite validation. Content analysis revealed five major themes: (1) Defining antibiotics, quality use of antibiotics and resistance, (2) antibiotic use: awareness and concern, (3) antimicrobial resistance: awareness and concern, (4) responding to antibiotic use and resistance, and (5) barriers to quality use of antibiotics and prevention of antibiotic resistance. The knowledge of quality use of antibiotics and resistance was promising, and the respondents were eager to address the drastic situation. The respondents were aware of the critical situation and provided valuable insights that can offer valued input while promoting the quality use of antibiotics in a developing country. The current study managed to identify an adequate understanding of antibiotic use and resistance among hospital pharmacists. Additionally, prospective concerns and possible predictors of antibiotic resistance were also highlighted. The current findings must be disseminated to the policymakers and prescribers to take prompt restorative actions to address antibiotic use and the development of antibiotic resistance in a developing country like Pakistan.

## 1. Introduction

The golden era of antibiotics regrettably did not last long as mankind was faced with the development of bacterial resistance against antibiotics [1]. Although Fleming, in his Nobel lecture, warned mankind by stating “it is not difficult to make microbes resistant to penicillin in the laboratory by exposing them to concentrations not sufficient to kill them” [2], minimum efforts were reported in the mid of 19th century to overcome antibiotic resistance [3]. Today, antibiotic resistance causes major risks to global safety and public health along with substantial societal impacts in the developing and developed world [4]. Kraker and associates in 2016 estimated that 10 million people will die due to antibiotic resistance by 2050 if restorative measures are not taken immediately [5]. Within this context, antibiotic resistance is driven by the inappropriate use of antibiotics in settings (hospitals and community) where antibiotics are not indicated, where guidelines for antibiotic use are not followed, or are considered clinically unnecessary for use among humans and animals [6]. Socioeconomic factors, self-medication, personal referrals, free availability of antibiotics, and unnecessary demands of the patients are also strong predictors of developing antibiotic resistance [7]. In a nutshell, antibiotic resistance is a multifactorial phenomenon that needs an imperative holistic approach and collaborative efforts of healthcare professionals, civil society, and community members to overcome the hazards presented to mankind and the generations to come.

Improving the quality use of antibiotics in hospitals and other healthcare settings in addition to limiting use in agriculture is a crucial area to safeguard antibiotics for future generations. In line with what is being discussed, the World Health Organization highlights the significant role of healthcare professionals in limiting the frequency of antibiotic resistance [8]. Healthcare professionals can reduce antibiotic resistance rates through evidence-based prescribing and adopting quality use of antibiotics. Moreover, being an educator, healthcare professionals are involved in reporting antibiotic-resistant infections to surveillance teams and educating patients and community members regarding antibiotic resistance and the hazards of misuse of antibiotics [8]. Today, the involvement of healthcare professionals in promoting the quality use of antibiotics is more inevitable than ever as the COVID-19 pandemic brought an alarming increase in antibiotic resistance. The Centers for Disease Control and Prevention reported a 15% increase in resistant infections from 2019 to 2020 among seven major pathogens because of a rush of antibiotic use while dealing with COVID-19 [9]. Therefore, controlling antibiotic resistance and promoting the quality use of antibiotics is a moral, ethical, and professional obligation of healthcare professionals.

Among healthcare professionals, hospital pharmacists occupy a conspicuous position in reducing the rates of antibiotic resistance [10]. Our claims are supported by the published literature where hospital pharmacists and their involvement during therapeutic plan development had a substantial positive effect on the healthcare system and disease management [11,12]. Shifting our concerns to the role of hospital pharmacists and antibiotic resistance, Sakeena and colleagues in their narrative review reported that aptly trained hospital pharmacists, when integrated into the health care system, can make a significant impact in minimizing inappropriate antibiotic use and resistance [13]. Hospital pharmacists promote the safe and cost-effective use of antibiotics, and this is acknowledged by the global healthcare systems [14,15,16,17]. However, such data are reported frequently from the developed world, and this is a major limitation faced by healthcare and social scientists around the globe. Developing countries have not yet implemented pharmacist-led initiatives whereby hospital pharmacists can play a key role in minimizing unnecessary prescribing of antibiotics and developing local prescribing guidelines according to diagnoses and local antibiotic susceptibility patterns [13]. Therefore, we strongly advocate using the expertise of hospital pharmacists as medicine counsellors to rationalize antibiotic use in the developing world.

Parallel to the published literature, the role of the hospital pharmacist in disease management and clinical decision-making is shadowed in a developing country like Pakistan. There is a paucity of data on hospital pharmacists’ integration into the healthcare system. Moreover, the capability and proficiency of a hospital pharmacist are also questioned by other healthcare professionals. Based on this dearth of information, we aimed to evaluate how hospital pharmacists view the quality use of antibiotics and antibiotic resistance practicing at a local healthcare facility in Quetta city, Pakistan. Conducting this study had twofold reasons: to generate data that can be used as a potential reference for further studies, and to highlight what hospital pharmacists can offer while dealing with antibiotic resistance.

## 2. Results

### 2.1. Demographic Information

The demographics are given in Table 1. Fifteen participants were approached; however, three refused participations because of their busy schedule. Therefore, interviews were conducted with twelve participants. Although the saturation was reached at the 10th interview, two additional interviews were carried out for absolute validation.

Most of the participants were male (75%), with age ranging from 25 to 35 years (80%). Half of the pharmacists had a Doctor of Pharmacy degree and had 11–20 years of experience.

**Figure 1:**
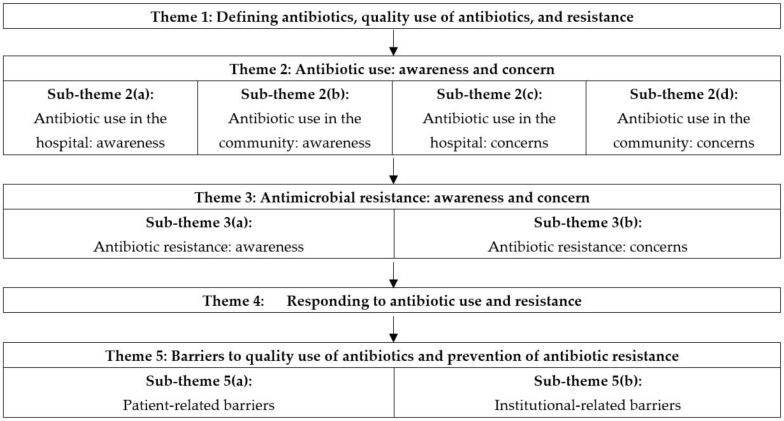
Schematic presentation of themes and sub-themes identified during data analysis.

The thematic content analysis revealed five themes and eight subthemes which are shown in Figure 1.

### 2.2. Theme 1: Defining Antibiotics, Quality Use of Antibiotics, and Resistance

As expected, all pharmacists had a clear understanding of antibiotics, the quality use of antibiotics, and the development of antibiotic resistance. Additionally, the pharmacists mentioned routinely updating their knowledge about antibiotic use and resistance through updated information sources (journal articles, trusted websites, and books). This is encouraging because evidence-based information in pharmacy practice incorporates pharmacists’ clinical expertise with the most accessible evidence. The availability of evidence-based information also helps in justifying the medication-related needs of the healthcare system, prescribing practices, and patients’ predilections [18,19].

“Antibiotics; Fleming’ gift for mankind are static and cidal in nature. These are the drugs of choice against primary and secondary bacterial infections.” (Pharmacist 1)

In parallel, pharmacists had decent knowledge about antibiotic resistance. It was obvious that pharmacists understood the phenomenon that can eventually help in the delivery of pharmaceutical care [20].

“The over-use and misuse of antibiotics, bacterial mutations, and substantial use of antibiotics in agriculture and among animals (like poultry and livestock) result in developing antibiotic resistance.” (Pharmacist 3)

### 2.3. Theme 2: Antibiotic Use: Awareness and Concern

#### 2.3.1. Sub-Theme 2(a): Antibiotic Use in the Hospital: Awareness

Respondents of the current study acknowledged frequent use of antibiotics at the setting site. Furthermore, as the influx of inpatients is high compared to outpatients, the use of parenteral antibiotics was commonly reported. Among the commonly prescribed antibiotics were Ceftriaxone (3rd generation cephalosporin), Ciprofloxacin (fluoroquinolones), Vancomycin (glycopeptides), Meropenem (carbapenem), and Piperacillin-Tazobactam (β-lactam/beta-lactamase inhibitors).

“The physicians prescribe oral antibiotics to the outpatients (based on the availability in the central pharmacy); however, Meropenem and Vancomycin are frequently used (inpatient) when compared to other antibiotics. It is estimated that every third or maybe fourth prescription contains these two drugs.” (Pharmacist 6)

#### 2.3.2. Sub-Theme 2(b): Antibiotic Use in the Community: Awareness

The absence of an effective surveillance system and poorly regulated legislature results in the free availability of antibiotics without prescription. Community pharmacies (medical stores) lack authorized personnel (community pharmacists) and are operated by laymen with no prior knowledge, qualification, or training in running a community pharmacy. Most of the pharmacists had information about antimicrobial dispensing rules according to Pakistan’s Drug Act 1976 and the Drug Regulatory Authority of Pakistan Act 2012. However, few know the drug laws (schedule G and D) that elaborate the use and dispensing of antibiotics at the communal level.

“From brands to generics, everything is freely available at the pharmacies. Everybody knows about it including policymakers and officials of the inspection teams. Till today, no one took serious action against the free availability and public sale of antibiotics.” (Pharmacist 7)

#### 2.3.3. Sub-Theme 2(c): Antibiotic Use in the Hospital: Concerns

The limited availability of antibiotics in the hospital and a high influx of patients requiring antibiotics was reported as a significant concern by all pharmacists. This is reasonable as the healthcare facilities are limited (in terms of human resources, utilities, and funding) and the prescribers have no or limited choice to prescribe antibiotics based on the availability in the central pharmacy.

“If I can recall, there are not more than 10 antibiotics available in the hospital. Therefore, antibiotic selection is based on availability and not on therapeutic needs. Rationally, yes this is malpractice, but what other choice do we have?” (Pharmacist 10)

#### 2.3.4. Sub-Theme 2(d): Antibiotic Use in the Community: Concerns

The free availability of antibiotics, antibiotic sharing, and nonprofessional referrals of antibiotic use in the community was the primary concern of the pharmacists. These factors were linked to economic and therapeutic loss to society. Moreover, this free availability and frequent use of antibiotics were rated as important factors in developing antibiotic resistance. In comparison, antibiotic use in the community was ranked as a substantial problem when compared to antibiotic use in hospitals.

“Just name the antibiotic, the quantity and there you have it. There are zero concepts of the recommended dosage, treatment duration, and actual need for antibiotics. Community pharmacies are encouraging antibiotic resistance, and this is increasing day by day.” (Pharmacist 8)

### 2.4. Theme 3: Antimicrobial Resistance: Awareness and Concern

#### 2.4.1. Sub-Theme 3(a): Antibiotic Resistance: Awareness

The respondents agreed that antibiotic resistance is a significant issue and has increased after the pandemic. They were also of the opinion that the irrational use of antibiotics at the communal level is promoting antibiotic resistance and needs imperative attention.

“Irrational prescribing, self-medication, using leftovers, all results in antibiotic resistance. Other reasons are also reported in the literature, but we have to admit that the issue is serious and needs prompt actions.” (Pharmacist 4)

#### 2.4.2. Sub-Theme 3(b): Antibiotic Resistance: Concerns

“Last week a six-month child was subjected to cultural sensitivity. The kid was resistant to eleven tested antibiotics.” (Pharmacist 5). The development of antibiotic resistance was taken seriously by all respondents that are resulting in suffering, deaths, and economic loss. Pharmacists also reported that antibiotic resistance at their practicing site is also emerging as treatment failure is often reported with the use of first or second-line antibiotics.

“I observed that compared to last year, antibiotics (specifically Ciprofloxacin and Ceftriaxone) are least effective. The physicians are now routinely prescribing Meropenem, Vancomycin, and Tazobactam. We must wake up because this is a serious concern and as I see it, there is no solution in near future too.” (Pharmacist 1)

### 2.5. Theme 4: Responding to Antibiotic Use and Resistance

As evident from the above conversation, our respondents were versed in antibiotic use and resistance. Consequently, we wanted to extract pharmacists’ viewpoints on how they respond to antibiotic use and the development of antibiotic resistance in their practice settings. Although all respondents agreed that they play a pivotal role in medicine management, mixed views were observed when the response to antibiotic use was investigated.

“Although I follow need and evidence-based medication (specifically when it comes to antibiotic), I must keep an eye on the generic availability in our stock.” (Pharmacist 9)

Six pharmacists claimed that they often try to convince the patients about generic substitution to save costs as well as about the importance of completing the complete therapy. Most of the patients come from a meager income background; they have no idea about the quality use of antibiotics.

“I normally guide the patients about the importance of antibiotics, the hazards of antibiotic resistance, and the financial and social repercussions. I hope that a medically educated patient can help in halting the development of antibiotic resistance.” (Pharmacist 11)

Three of the interviewees agreed that we could not control antibiotic resistance alone without implementing guidelines, laws, and legislation for antibiotic use. The interviewed hospital pharmacists also pointed out a few strategies to control the emergence of antibiotic resistance by involving all key stakeholders of the healthcare system.

“Alone, we cannot reduce antibiotic resistance. It is emerging at a high pace and a collective approach is needed to overcome this problem. The policymakers should target a mass population as well as an individualized strategy that must focus on community members and healthcare professionals to safeguard the use of antibiotics.” (Pharmacist 9)

### 2.6. Theme 5: Barriers to Quality Use of Antibiotics and Prevention of Antibiotic Resistance

It is now acknowledged that antibiotic resistance can be reduced by prescribing antibiotics rationally based on established guidelines, antibiotic susceptibility testing, and clinical response. In parallel, the surveillance of antibiotic availability in healthcare settings, restrictive self-medication, and over-prescription is also needed. However, antibiotic resistance can only be controlled and minimized by the determined efforts of all healthcare professionals (physicians, pharmacists, nurses). Several barriers were identified by the study respondents while addressing the quality use of antibiotics and the development of antibiotic resistance and will be discussed consequently.

#### 2.6.1. Subtheme 5(a): Patient-Related Barriers

Self-medication, using leftover antibiotics, referral of antibiotics (friends and families), expecting an antibiotic during the consultation, and demanding an antibiotic by themselves were identified as the key barriers to the quality use of antibiotics and antibiotic resistance. Patient education and counseling were last reported at the healthcare institutes and that was rated as a major factor in developing the false ideology about antibiotics as mentioned above.

“Our patients demand antibiotics and will go to different stores to get one. The physician is considered incompetent if an antibiotic is not prescribed. This mindset is shaping as a key barrier to quality use of antibiotics in our society.” (Pharmacist 3)

Another barrier to rational use of antibiotics was related to the urgency of being cured. We must agree that when faced with a disease, expecting a quick recovery is obvious. However, this tendency does not allow misuse of antibiotics considering that it will pace up the recovery time. Nevertheless, patients are in the habit of using antibiotics for a fast pace of recovery, and this is shaping as a factor in developing antibiotic resistance.

“While being questioned (by physicians or nurses), using an antibiotic before coming to the hospital is usually reported by the patients. The reason is always the same (it cures everything). This is an issue that we are facing almost daily. Don’t you think this is causing antibiotic resistance?” (Pharmacist 4)

#### 2.6.2. Subtheme 5(a): Institutional-Related Barriers

Most of the respondents also emphasized certain institution-related deficiencies and limitations as barriers to the quality use of antibiotics and antibiotic resistance. The most reported barrier was the limited number of antibiotics available at the central pharmacy. Medicines are supplied annually based on hospital demand and because purchasing budgets are low, medicines are not adequately available. The availability is attained by reducing the quantity and types of medicines (when one class of antibiotic is available, the other class is inevitably rejected). However, this limits the choice of the prescribers, and they must prescribe what is available at the hospital.

“We work in a public hospital where 70–80% of medicine is provided by the hospital to the inpatients. However, we have financial limitations, and availability of antibiotics from all therapeutic classes is not possible.” (Pharmacist 12)

Another barrier to the quality use of antibiotics was related to the limited capacity of cultural sensitivity. By practice, the developed guidelines advocate an initiation of empirical therapy followed by a culture sensitivity test. However, this was last performed at the study site, and without sensitivity reports being available, the practice promoted antibiotic resistance.

“We have limited the capacity of performing a culture sensitivity test. Because most of our patients belong to the below-average income group, ordering a sensitivity test is unaffordable for the patients. In such scenarios, we have no choice but to continue using the same antibiotics.” (Pharmacist 10)

## 3. Discussion

For decades, healthcare and social scientists have been trying to minimize the burden of antibiotic resistance. Among those, numerous interventions and measures have been taken that too have reported their effectivity and effectuality [21,22,23,24]. However, with the development of new resistance mechanisms, antibiotic resistance is rising dangerously around the globe [8]. Additionally, the emergence of COVID-19 reported increased use of antibiotics that again resulted in augmenting antibiotic resistance [25,26]. Hence, the role of healthcare professionals in promoting the quality use of antibiotics remains crucial as they are the frontline specialists while tackling antibiotic resistance and its consequences. Among healthcare professionals, because of their duty for rationalizing antibiotic use, being medicine managers and patient educators, hospital pharmacists are critically placed and can play a key role in promoting the safe use of antibiotics. Within this context, we are aware that literature does mention hospital pharmacists’ viewpoint on antibiotic use and resistance [27,28,29,30], but nothing is reported from the current study settings. We are also aware that several studies on the knowledge and practices of pharmacists are reported from Pakistan, but the target audience was different from what is reported in our study. Where Saleem et al. focused community pharmacists [31], Mubarak et al. targeted pharmacy students regarding their views of antibiotic use and resistance [32] and widely held information focused on antibiotic stewardship. Therefore, one distinct advantage of the current study is the pioneer study from Pakistan that evaluated the knowledge and attitudes of hospital pharmacists about antibiotic use and resistance along with the views and concerns on contributing factors.

Pharmacists’ positive perception towards the quality use of antibiotics and antibiotic resistance was an encouraging finding of the current study. However, mixed observations were reported when the results of the current study were cross-compared with the published literature. Al-Tanni et al. in their study concluded that although pharmacists’ knowledge of the quality use of antibiotics was satisfactory, the perception towards the spread of resistance was unsatisfactory [33]. Similarly, Tang et al. in their multi-centered study also identified significant knowledge gaps among pharmacists and the gap was prominent among work settings [34]. The European Centre for Disease Prevention and Control (ECDC) surveyed 1204 hospital pharmacists in 2019 and reported that although the respondents had good knowledge of antibiotics, ensuring the knowledge about resistance was highlighted as an area of improvement among the pharmacists [35]. Better knowledge of the current study respondents is a positive indication when correlating it with the goal of the Institute of Medicine (IMS) suggested in 2020. Accordingly, IMS proposed that by 2020, 90% of clinical decisions must be supported with accurate, timely, and up-to-date clinical information that should reflect the best available evidence to achieve the best patient outcomes [36]. Hospital pharmacists of the current study contained updated information (revealed during informal discussion) and that is an indication of providing an effective therapeutic plan when it comes to the quality use of antibiotics and containment of antibiotic resistance.

The results of this qualitative study revealed that the use of broad-spectrum antibiotics is widespread at the study site. Our respondents were aware and anxious about this imprudent use of antibiotics, and this is similar to the findings of Tarrant et al. [37]. The authors also reported frequent use of broad-spectrum antibiotics in three countries, which is supported by studies of the same nature [33,38]. Among the drivers, the use of broad-spectrum antibiotics was also mentioned by the current study respondents. A method of overcoming these issues is establishing medical education programs and providing credits to the prescribers that can act as a benchmark in annual assessment plans.

Another key finding of the current study was the limited availability of antibiotics in the hospital and hence a reduced choice for the prescribers while dealing with infectious diseases. In addition, poor culture sensitivity testing was highlighted. Unfortunately, the evidence-based data are lacking from Baluchistan, and we do not have an actual picture of such limitations. However, our personal observation goes parallel to what is reported by the respondents as there is a lack or limited number of antibiotics at the hospital. Within this context, the healthcare system of Pakistan is faced with severe financial limitations and is unable to cater to the needs of the patients [39]. Healthcare financing in Pakistan is mainly out-of-pocket, there are inequities at the healthcare levels, and at times care is not attained because of non-affordability [39]. The pandemic crisis and the current financial crunch are again posing a great threat to medicine availability at public healthcare facilities [40]. Although the reasons are genuine, limited availability, continuous use of the same antibiotics, and lack of sensitivity testing are posing an incessant threat to the development of antibiotic resistance. Frankly speaking, we do not see a solution to this threat soon, but it is true that there is going to be a continuous rise in antibiotic resistance. Healthcare professionals, policymakers, and financial stakeholders must realize the severity of this issue and propose concrete measures to ensure the appropriate availability of antibiotics at public healthcare institutions. Our suggestions are in line with the recommendations of the Pakistan Antimicrobial Resistance Surveillance System Surveillance Report of 2020 whereby the limitations were acknowledged and immediate actions to overcome the limitations were advocated.

Finally, patient-reported factors were rated as a predictor of antibiotic resistance by the study respondents. Among these, self-medications, personal referrals, and demanding an antibiotic were the major variables. These findings are not new, and the literature does support the claims of our study respondents. Nepal and Bhatta in their systematic review highlighted the high prevalence of self-medication of antibiotics in the WHO Southeast Asian Region and this hence was the leading cause of antibiotic resistance [41]. Similarly, Väänänen and Airaksinen also reported excessive and nonsensical self-medication of antibiotics in the European region [42]. Correspondingly, Nair and colleagues in their qualitative study confirmed that patients tend to demand antibiotics as they seek a fast cure [43]. Such comparable findings indicate a lack of communal knowledge of antibiotic use and resistance and the need for immediate attention from healthcare providers, especially pharmacists. Patients are to be provided ample medical education regarding antibiotic use and resistance and pharmacists must step up to engage their patients in education and counselling. In addition, the policymakers must ensure strict compliance with antibiotic sales at the community level and employ mass educational strategies to halt self-medication and referral of suggesting antibiotics to a friend, family, and societal members.

## 4. Materials and Methods

### 4.1. Study Design and Settings

Because of the scarcity of information, a qualitative study design (in-depth, face-to-face interviews) was the ultimate choice. Being amenable, qualitative methods can extract attitudes, experiences, and intentions that are often missed in a quantitative phase [44,45]. Because the research team was faced with limited published literature, using a qualitative design was appropriate as it can generate a wide range of ideas and opinions and divulging viewpoints [46,47]. As we were aiming for inductive approaches to generate ideologies and concepts, a qualitative design was also adopted because it has more potential for research than any other models [48]. Additionally, the COREQ checklist was used to describe the qualitative methodology and that is presented as Appendix A.

The research was conducted at the Surgical Department of Sandeman Provincial Hospital, Quetta (SPHQ). Centrally located, SPHQ is a tertiary care hospital and provides generalized healthcare facilities. Additionally, being a public institute, SPHQ is a choice for most of the population [49].

### 4.2. Study Participants, Criteria, and Sampling

Hospital pharmacists stationed and practicing at the surgical unit and consenting to participate in the study were approached for data collection. Based on our objective, it was apparent to adopt the purposive sampling method [50]. Pharmacists on rotations and not willing to participate were excluded.

### 4.3. The Interview Guide (Validation, Reliability, and Pilot Study)

We constructed a semi-structured interview guide after an extensive literature review [51,52,53,54,55], through expert panel discussion and experience sharing [56,57,58]. To extract maximum information, we intentionally constructed the guide with widely framed, open-ended questions. Additionally, pharmacists were allowed to provide their narratives and to share information relevant to antibiotic use and resistance.

The guide was constructed in the English language and was subjected to face and content validity through a panel of experts (senior pharmacists). Once the validity was ensured, the guide was piloted with four pharmacists to ensure that the topics to be discussed were at the level that respondents would comprehend with ease. The preliminary data and conclusion confirmed that the discussion topics were enough and appropriately phrased to answer research questions and minimize validity and reliability threats. As the validity and reliability of the discussion guide were ensured, it was made available for the main study. Data and participants of the pilot study were not included in the final analysis.

### 4.4. Interview Procedure, Data Collection, and Analysis

ZI (male, a certified medical practitioner, District Health Officer, certified in Qualitative methods) and FS (male, academic pharmacist with a PhD having experience of qualitative research with numerous published articles) conducted the interviews. FS was also involved in carrying out field notes during the interviews.

Interviews were conducted at the pharmacist’s office in the surgical unit. All participants were briefed about the study objectives before the interviews. A debriefing session was again conducted at the end of the discussion. The interviews started with an ice-breaking session. Probing questions were asked in between conversations to clarify the meanings of responses and to gain insight into the topic being discussed.

The phenomenology-based approach commissioned in-depth, face-to-face interviews. All interviews were audio-taped followed by transcribed verbatim and were then analyzed for thematic contents by the standard content analysis framework. Each interview lasted for approximately half an hour. To draw in-depth views, the freedom to express additional reviews and comments was given. Interviews were conducted until thematic saturation was reached [59,60]. No repeat interviews were carried out.

The research team analyzed the recordings (verbatim) and later arranged an informal gathering where pharmacist were presented with the finalized interview scripts [61]. They were asked for confirmation of the precision and accuracy of words, ideas, and jargon used during the script analysis. Once confirmed, the transcripts were subjected to thematic content analysis whereby four data coders were involved in the process [62,63]. NVivo ^®^ was used for coding and analysis through iterations [64] and inconsistencies were resolved through mutual consensus. Interviews were coded line-by-line, and an initial list of nodes was developed. Later, this augmented in developing the framework and transcripts were coded accordingly. New emerging nodes were added to the existing list and were categories as emerging themes. All emerging themes and subthemes were discussed among the research team for accuracy and were presented for data inference and interpretation.

## 5. Conclusions

The current study managed to identify an adequate understanding of antibiotic use and resistance among hospital pharmacists. Additionally, prospective concerns (limited availability of antibiotics) and possible predictors of antibiotic resistance (lack of sensitivity testing, self-medication, referrals, and demanding an antibiotic) were also highlighted factors contributing factors were also identified. As corrective measures, respondents of the current study focused on strict legislation to overcome the free availability and sales of antibiotics at the community level, ensuring the implementation of guidelines for the prescribers and copious availability of antibiotics and sensitivity testing facilities at the healthcare institutes. Therefore, we urge that these findings must be disseminated to policymakers and prescribers to take restorative action as soon as possible. Antibiotic resistance is a global threat, and we need a holistic approach to tackle this issue for generations while combating infectious diseases with precision.

## 6. Limitations and Recommendations

Qualitative methods have their limitations. Although we ensured saturation, the convenience sampling approach does not offer views of the participants that are not interviewed. Likewise, generalizability is always an issue with qualitative methods. However, with rich data extraction, we are confident that the study has provided baseline information for prospective studies. Therefore, we are recommending an in-depth and detailed study (quantitative) focusing on a large cohort of hospital pharmacists to ensure the validity of the current findings.

## Figures and Tables

**Table 1:** Demographic characteristics of the pharmacists.

Demographics	Frequency	Percentage
Gender		
Male	9	75%
Female	3	25%
Age		
25–35	10	80%
36–50	2	20%
Qualification		
Doctor of Pharmacy	7	58.3%
M.Phil	4	33.3%
Ph.D.	1	8.4%
Experience in years		
1–10 years	5	41.6%
11–20 years	7	58.4%
Designation		
Hospital pharmacist	11	91.6%
Chief pharmacist	1	8.4%

## Data Availability

The data are available from the corresponding author upon reasonable request.

## Appendix A

**Table A1:** The consolidated criteria for reporting qualitative studies (COREQ).

Topic	Guide Questions/Description	Remarks	Page No.
Domain 1: Research team and reflexivity			
(a) Personal Characteristics			
Interviewer/facilitator	Which author/s conducted the interview or focus group?	Two authors ZI and FS conducted the interviews.	9
Credentials	What were the researcher’s credentials? e.g., Ph.D., MD	ZI: MBBS; MPH FS: Ph.D.	9
Occupation	What was their occupation at the time of the study?	ZI: Deputy District Health Officer, Government of Baluchistan FS: Academic/pharmacist	9
Gender	Was the researcher male or female?	ZI: Male FS: Male	9
Experience and training	What experience or training did the researcher have?	ZI: Certification in qualitative research methods (CQRM); attended workshop on NViVO for data analyses. FS: an experienced researcher in qualitative studies and has published numerous qualitative research articles.	9
(b) Relationship with participants			
Relationship established	Was a relationship established prior to study commencement?	The relationship was developed only for the current study.	N/A
Participant knowledge of the interviewer	What did the participants know about the researcher? e.g., personal goals, reasons for doing the research	None of the participants knew about the researchers.	N/A
Interviewer characteristics	What characteristics were reported about the inter viewer/facilitator? E.g., Bias, assumptions, reasons, and interests in the research topic	The characteristics were presented as researchers and authors.	1
Domain 2: Study design			
(a) Theoretical framework			
Methodological orientation and Theory	What methodological orientation was stated to underpin the study? e.g., grounded theory, discourse analysis, ethnography, phenomenology, content analysis	Phenomenology and thematic content analysis.	9
(b) Participant selection			
Sampling	How were participants selected? e.g., purposive, convenience, consecutive, snowball	The participants were purposively selected.	9
Method of approach	How were participants approached? e.g., face-to-face, telephone, mail, email	The participants were approached face-to-face.	9
Sample size	How many participants were in the study?	12 participants were approached for the interviews.	3
Non-participation	How many people refused to participate or dropped out? Reasons?	We approached 15 participants. Three people refused as they were busy with their routine work.	3
(c) Setting			
Setting of data collection	Where was the data collected? e.g., home, clinic, workplace	Data were collected at the pharmacists’ workplace.	9
Presence of non-participants	Was anyone else present besides the participants and researchers?	No-one else was present to ensure confidentiality of the responses.	N/A
Description of sample	What are the important characteristics of the sample? e.g., demographic data, date.	The important characteristics are presented in Table 1.	3
(d) Data collection			
Interview guide	Were questions, prompts, guides provided by the authors? Was it pilot tested?	A semi-structured interview guide was developed, and pilot tested with 4 pharmacists. Data of the pilot phase was not included in the final analysis.	9
Repeat interviews	Were repeat inter views carried out? If yes, how many?	No repeat interviews were carried.	9
Audio/visual recording	Did the research use audio or visual recording to collect the data?	All interviews were audio recorded.	9
Field notes	Were field notes made during and/or after the interview or focus group?	FS prepared then field notes during the interviews that assisted the transcription.	9
Duration	What was the duration of the inter views or focus group?	The duration of the in-depth interviews was approximately 30 min.	9
Data saturation	Was data saturation discussed?	Yes	3
Transcripts returned	Were transcripts returned to participants for comment and/or correction?	Yes, transcripts were return for confirmation of the precision and accuracy of words, ideas, and jargon used during the script analysis.	9
Domain 3: Analysis and findings			
(a) Data analysis			
Number of data coders	How many data coders coded the data?	Four data coders coded the data.	9
Description of the coding tree	Did authors provide a description of the coding tree?	Interviews were coded line-by-line, and an initial list of nodes was developed. Later, this augmented in developing the framework and transcripts were coded accordingly. New emerging nodes were added to the existing list and were categories as emerging themes.	9
Derivation of themes	Were themes identified in advance or derived from the data?	All themes were derived from the data.	4–7
Software	What software, if applicable, was used to manage the data?	NVivo^®^ was used to manage the data.	9
Participant checking	Did participants provide feedback on the findings?	No	N/A
(b) Reporting			
Quotations presented	Were participant quotations presented to illustrate the themes/findings? Was each quotation identified? e.g., participant number	Yes, all quotations were cross matched with the respondent’s demographics.	4–7
Data and findings consistent	Was there consistency between the data presented and the findings?	Yes	4–7
Clarity of major themes	Were major themes clearly presented in the findings?	Yes	4–7
Clarity of minor themes	Is there a description of diverse cases or discussion of minor themes?	Sub themes were identified and are presented and discussed in the manuscript.	4–7

## References

[1] Uddin T.M., Chakraborty A.J., Khusro A., Zidan B.R.M., Mitra S., Emran T.B., Dhama K., Ripon M.K.H., Gajdács M., Sahibzada M.U.K. (2021). Antibiotic resistance in microbes: History, mechanisms, therapeutic strategies and future prospects. J. Infect. Public Health.

[2] Action on Antibiotic Resistance The Discovery of Antibiotics. https://www.reactgroup.org/antibiotic-resistance/course-antibiotic-resistance-the-silent-tsunami/part-1/the-discovery-of-antibiotics/#:~:text=In%20his%20Nobel%20lecture%20in,occasionally%20happened%20in%20the%20body.

[3] Podolsky S.H. (2018). The evolving response to antibiotic resistance (1945–2018). Palgrave Commun..

[4] Ben Y., Fu C., Hu M., Liu L., Wong M.H., Zheng C. (2019). Human health risk assessment of antibiotic resistance associated with antibiotic residues in the environment: A review. Environ. Res..

[5] De Kraker M.E., Stewardson A.J., Harbarth S. (2016). Will 10 million people die a year due to antimicrobial resistance by 2050?. PLoS Med..

[6] Aslam B., Wang W., Arshad M.I., Khurshid M., Muzammil S., Rasool M.H., Nisar M.A., Alvi R.F., Aslam M.A., Qamar M.U. (2018). Antibiotic resistance: A rundown of a global crisis. Infect. Drug Resist..

[7] Ilić K., Jakovljević E., Škodrić-Trifunović V. (2012). Social-economic factors and irrational antibiotic use as reasons for antibiotic resistance of bacteria causing common childhood infections in primary healthcare. Eur. J. Pediatr..

[8] World Health Organization Antibiotic Resistance. https://www.who.int/news-room/fact-sheets/detail/antibiotic-resistance.

[9] Centers for Disease Control and Prevention COVID-19 Reverses Progress in Fight against Antimicrobial Resistance in U.S. https://www.cdc.gov/media/releases/2022/s0712-Antimicrobial-Resistance.html#:~:text=In%20the%20report%2C%20CDC%20analyzed,to%202020%20among%20seven%20pathogens.

[10] Khan N., McGarry K., Naqvi A.A., Iqbal M.S., Haider Z. (2020). Pharmacists’ viewpoint towards their professional role in healthcare system: A survey of hospital settings of Pakistan. BMC Health Serv. Res..

[11] Clay P.G. (2016). Turning the criticism into construction. J. Am. Pharm. Assoc..

[12] Greer N., Bolduc J., Geurkink E., Rector T., Olson K., Koeller E., MacDonald R., Wilt T.J. (2016). Pharmacist-led chronic disease management: A systematic review of effectiveness and harms compared with usual care. Ann. Intern. Med..

[13] Sakeena M., Bennett A.A., McLachlan A.J. (2018). Enhancing pharmacists’ role in developing countries to overcome the challenge of antimicrobial resistance: A narrative review. Antimicrob. Resist. Infect. Control.

[14] Ellis K., Rubal-Peace G., Chang V., Liang E., Wong N., Campbell S. (2016). Antimicrobial stewardship for a geriatric behavioral health population. Antibiotics.

[15] Okada N., Fushitani S., Azuma M., Nakamura S., Nakamura T., Teraoka K., Watanabe H., Abe M., Kawazoe K., Ishizawa K. (2016). Clinical evaluation of pharmacist interventions in patients treated with anti-methicillin-resistant *Staphylococcus aureus* agents in a hematological ward. Biol. Pharm. Bull..

[16] Yen Y.-H., Chen H.-Y., Wuan-Jin L., Lin Y.-M., Shen W.C., Cheng K.-J. (2012). Clinical and economic impact of a pharmacist-managed iv-to-po conversion service for levofloxacin in Taiwan. Int. J. Clin. Pharmacol. Ther..

[17] Zhou Y., Ma L.Y., Zhao X., Tian S.H., Sun L.Y., Cui Y.M. (2015). Impact of pharmacist intervention on antibiotic use and prophylactic antibiotic use in urology clean operations. J. Clin. Pharm. Ther..

[18] Al-Quteimat O.M., Amer A.M. (2016). Evidence-based pharmaceutical care: The next chapter in pharmacy practice. Saudi Pharm. J..

[19] Lewis S.J., Orland B.I. (2004). The importance and impact of Evidence Based Medicine. J. Manag. Care Pharm..

[20] Chan A.H.Y., Beyene K., Tuck C., Rutter V., Ashiru-Oredope D. (2022). Pharmacist beliefs about antimicrobial resistance and impacts on antibiotic supply: A multinational survey. JAC Antimicrob. Resist..

[21] Borek A.J., Campbell A., Dent E., Butler C.C., Holmes A., Moore M., Walker A.S., McLeod M., Tonkin-Crine S. (2021). Implementing interventions to reduce antibiotic use: A qualitative study in high-prescribing practices. BMC Fam. Pract..

[22] Raban M.Z., Gasparini C., Li L., Baysari M.T., Westbrook J.I. (2020). Effectiveness of interventions targeting antibiotic use in long-term aged care facilities: A systematic review and meta-analysis. BMJ Open.

[23] Rogers Van Katwyk S., Grimshaw J.M., Nkangu M., Nagi R., Mendelson M., Taljaard M., Hoffman S.J. (2019). Government policy interventions to reduce human antimicrobial use: A systematic review and evidence map. PLoS Med..

[24] Smith R.D., Coast J., Millar M.R., Wilton P., Karcher A.-M. (2001). Interventions against Antimicrobial Resistance: A Review of the Literature and Exploration of Modelling Cost-Effectiveness. Global Forum for Health Research.

[25] Getahun H., Smith I., Trivedi K., Paulin S., Balkhy H.H. (2020). Tackling antimicrobial resistance in the COVID-19 pandemic. Bull. World Health Organ..

[26] Hashmi F.K., Atif N., Malik U.R., Saleem F., Riboua Z., Hassali M.A., Butt M.H., Mallhi T.H., Khan Y.H. (2022). In pursuit of COVID-19 treatment strategies: Are we triggering antimicrobial resistance?. Disaster Med. Public Health Prep..

[27] Broom A., Broom J., Kirby E., Plage S., Adams J. (2015). What role do pharmacists play in mediating antibiotic use in hospitals? A qualitative study. BMJ Open.

[28] Broom A., Broom J., Kirby E., Scambler G. (2015). The path of least resistance? Jurisdictions, responsibility and professional asymmetries in pharmacists’ accounts of antibiotic decisions in hospitals. Soc. Sci. Med..

[29] Dooling K.L., Kandeel A., Hicks L.A., El-Shoubary W., Fawzi K., Kandeel Y., Etman A., Lohiniva A.L., Talaat M. (2014). Understanding antibiotic use in Minya District, Egypt: Physician and pharmacist prescribing and the factors influencing their practices. Antibiotics.

[30] Garau J., Bassetti M. (2018). Role of pharmacists in antimicrobial stewardship programmes. Int. J. Clin. Pharm..

[31] Saleem Z., Hassali M.A., Hashmi F.K., Godman B., Saleem F. (2019). Antimicrobial dispensing practices and determinants of antimicrobial resistance: A qualitative study among community pharmacists in Pakistan. Fam. Med. Community Health.

[32] Mubarak N., Arif S., Irshad M., Aqeel R.M., Khalid A., Ijaz U.E.B., Mahmood K., Jamshed S., Zin C.S., Saif-Ur-Rehman N. (2021). How Are We Educating Future Physicians and Pharmacists in Pakistan? A Survey of the Medical and Pharmacy Student’s Perception on Learning and Preparedness to Assume Future Roles in Antibiotic Use and Resistance. Antibiotics.

[33] Al-Taani G.M., Al-Azzam S., Karasneh R.A., Sadeq A.S., Mazrouei N.A., Bond S.E., Conway B.R., Aldeyab M.A. (2022). Pharmacists’ Knowledge, Attitudes, Behaviors and Information Sources on Antibiotic Use and Resistance in Jordan. Antibiotics.

[34] Tang K.L., Teoh T.F., Ooi T.T., Khor W.P., Ong S.Y., Lim P.P., Abdul Karim S., Tan S.S.A., Ch’ng P.P., Choong Y.C. (2020). Public hospital pharmacists’ perceptions and knowledge of antibiotic use and resistance: A multicenter survey. Antibiotics.

[35] Ashiru-Oredope D., Hopkins S., Vasandani S., Umoh E., Oloyede O., Nilsson A., Kinsman J., Elsert L., Monnet D.L. (2021). Healthcare workers’ knowledge, attitudes and behaviours with respect to antibiotics, antibiotic use and antibiotic resistance across 30 EU/EEA countries in 2019. Eurosurveillance.

[36] McGinnis J.M., Goolsby W.A., Olsen L. (2009). Leadership Commitments to Improve Value in Health Care: Finding Common Ground: Workshop Summary.

[37] Tarrant C., Colman A.M., Jenkins D.R., Chattoe-Brown E., Perera N., Mehtar S., Nakkawita W.D., Bolscher M., Krockow E.M. (2021). Drivers of Broad-Spectrum Antibiotic Overuse across Diverse Hospital Contexts—A Qualitative Study of Prescribers in the UK, Sri Lanka and South Africa. Antibiotics.

[38] Lubwama M., Onyuka J., Ayazika K.T., Ssetaba L.J., Siboko J., Daniel O., Mushi M.F. (2021). Knowledge, attitudes, and perceptions about antibiotic use and antimicrobial resistance among final year undergraduate medical and pharmacy students at three universities in East Africa. PLoS ONE.

[39] Malik M.A., Wasay M. (2013). Economics of health and health care in Pakistan. J. Pak. Med. Assoc..

[40] The Express Tribune ‘Pakistan in Financial Emergency’. https://tribune.com.pk/story/2389724/pakistan-in-financial-emergency.

[41] Nepal G., Bhatta S. (2018). Self-medication with antibiotics in WHO Southeast Asian Region: A systematic review. Cureus.

[42] Väänänen M.H., Pietilä K., Airaksinen M. (2006). Self-medication with antibiotics—Does it really happen in Europe?. Health Policy.

[43] Nair M., Tripathi S., Mazumdar S., Mahajan R., Harshana A., Pereira A., Jimenez C., Halder D., Burza S. (2019). Knowledge, attitudes, and practices related to antibiotic use in Paschim Bardhaman District: A survey of healthcare providers in West Bengal, India. PLoS ONE.

[44] Berg B.L., Lune H., Lune H. (2004). Qualitative Research Methods for the Social Sciences.

[45] Kitzinger J. (1995). Qualitative research: Introducing focus groups. Br. Med. J..

[46] Krueger R.A. (2009). Focus Groups: A Practical Guide for Applied Research.

[47] Stewart D.W., Shamdasani P.N. (2014). Focus Groups: Theory and Practice.

[48] Entwistle V.A., Renfrew M.J., Yearley S., Forrester J., Lamont T. (1998). Lay perspectives: Advantages for health research. Br. Med. J..

[49] Shahzad F., Saleem F., Iqbal Q., Haque N., Haider S., Salman M., Masood I., Hassali M.A., Iftikhar S., Bashaar M. (2018). A cross-sectional assessment of health literacy among hypertensive community of Quetta City, Pakistan. Biomed. J..

[50] Brace-Govan J. (2004). Issues in snowball sampling: The lawyer, the model and ethics. Qual. Res. J..

[51] Asante K.P., Boamah E.A., Abdulai M.A., Buabeng K.O., Mahama E., Dzabeng F., Gavor E., Annan E.A., Owusu-Agyei S., Gyansa-Lutterodt M. (2017). Knowledge of antibiotic resistance and antibiotic prescription practices among prescribers in the Brong Ahafo Region of Ghana; a cross-sectional study. BMC Health Serv. Res..

[52] Brooks L., Shaw A., Sharp D., Hay A.D. (2008). Towards a better understanding of patients’ perspectives of antibiotic resistance and MRSA: A qualitative study. Fam. Pract..

[53] Krockow E., Colman A., Chattoe-Brown E., Jenkins D., Perera N., Mehtar S., Tarrant C. (2019). Balancing the risks to individual and society: A systematic review and synthesis of qualitative research on antibiotic prescribing behaviour in hospitals. J. Hosp. Infect..

[54] Moongtui W., Picheansathian W., Senaratana W. Role of Nurses in Prevention of Antimicrobial Resistance. http://origin.searo.who.int/publications/journals/regional_health_forum/media/2011/V15n1/rhfv15n1p104.pdf.

[55] Nair M., Tripathi S., Mazumdar S., Mahajan R., Harshana A., Pereira A., Jimenez C., Halder D., Burza S. (2019). “Without antibiotics, I cannot treat”: A qualitative study of antibiotic use in Paschim Bardhaman district of West Bengal, India. PloS ONE.

[56] Kallio H., Pietilä A.M., Johnson M., Kangasniemi M. (2016). Systematic methodological review: Developing a framework for a qualitative semi-structured interview guide. J. Adv. Nurs..

[57] Morris A. (2015). A Practical Introduction to In-Depth Interviewing.

[58] Voutsina C. (2018). A practical introduction to in-depth interviewing. Int. J. Res. Method Educ..

[59] Nelson J. (2017). Using conceptual depth criteria: Addressing the challenge of reaching saturation in qualitative research. Qual. Res..

[60] Saunders B., Sim J., Kingstone T., Baker S., Waterfield J., Bartlam B., Burroughs H., Jinks C. (2018). Saturation in qualitative research: Exploring its conceptualization and operationalization. Qual. Quant..

[61] Guest G., MacQueen K.M., Namey E.E. (2012). Introduction to applied thematic analysis. Appl. Themat. Anal..

[62] Anderson R. (2007). Thematic content analysis (TCA). Descr. Present. Qual. Data.

[63] Vaismoradi M., Turunen H., Bondas T. (2013). Content analysis and thematic analysis: Implications for conducting a qualitative descriptive study. Nurs. Health Sci..

[64] Edhlund B., McDougall A. (2019). Nvivo 12 Essentials.

